# Respiratory tract antimicrobial peptides more effectively killed multiple methicillin-resistant *Staphylococcus aureus* and nontypeable *Haemophilus influenzae* isolates after disruption from biofilm residence

**DOI:** 10.1128/spectrum.03066-24

**Published:** 2025-06-18

**Authors:** Nikola Kurbatfinski, Joseph A. Jurscisek, Kathryn Q. Wilbanks, Steven D. Goodman, Lauren O. Bakaletz

**Affiliations:** 1 Center for Microbial Pathogenesis, Abigail Wexner Research Institute at Nationwide Children’s Hospital, Columbus, Ohio, USA; 2 Department of Pediatrics, The Ohio State University College of Medicine 12305 , Columbus, Ohio, USA; Reichman University, Herzeliya, Israel

**Keywords:** DNABII, NRel, LL-37, cathelicidin, beta defensins, MRSA, nontypeable *Haemophilus influenzae*

## Abstract

Bacteria newly released (NRel) from biofilm residence via multiple methodologies are commonly significantly more sensitive to antibiotics. We’ve induced NRel with this phenotype after incubation of biofilms formed by diverse human pathogens with an epitope-targeted monoclonal antibody directed at protective domains within bacterial DNABII proteins that provide structural support to the eDNA-dependent biofilm matrix. The observed heightened sensitivity was due, in part, to increased NRel membrane permeability. In three animal models of human biofilm-mediated infections, this monoclonal induced biofilm disruption with rapid concomitant bacterial clearance and disease resolution in the absence of any co-delivered antibiotic, which suggested a key role of innate immune effectors. Recently, we showed that NRel of the respiratory pathogen nontypeable *Haemophilus influenzae* (NTHI), as mediated by the DNABII-directed monoclonal, are also highly vulnerable to killing by human polymorphonuclear neutrophils (PMNs). Here, we extended these observations to show that the transient, yet highly vulnerable anti-DNABII NRel phenotype of three isolates of both NTHI and methicillin-resistant *Staphylococcus aureus* (MRSA) included significant sensitivity to killing by three antimicrobial peptides commonly expressed within the respiratory tract or by PMNs (e.g., human β-defensins 1 and 3 as well as the cathelicidin, LL-37). We envision induction of the NRel phenotype by delivery of this monoclonal antibody to patients with recalcitrant biofilm-mediated diseases to provide greatly improved medical management. Ideally, clearance of NRel will be mediated by innate immune effectors of an immunocompetent host or, if needed, by co-delivered traditional antibiotics, which are canonically ineffective against biofilm-resident bacteria but would be highly effective against NRel.

**IMPORTANCE:**

Pathogenesis of most common chronic and/or recurrent bacterial diseases (e.g., middle ear infections, urinary tract infections, rhinosinusitis, among others) can be attributed to biofilms that are canonically highly resistant to both immune effectors and antibiotics. If we treat biofilms formed by diverse human pathogens with a targeted monoclonal antibody directed at protective domains of bacterial DNA-binding proteins integral to the structural stability of the eDNA-rich biofilm matrix, they are rapidly disrupted with concomitant release of the resident bacteria. These newly released (NRel) bacteria are transiently significantly more sensitive to killing by both traditional antibiotics and human PMNs, and herein, we showed that they are also more readily killed by antimicrobial peptides. Clinically, we hope to leverage this understanding of the NRel phenotype for better medical management of these challenging infections, as well as perhaps even limit or eliminate further contribution to the global antimicrobial resistance ’pandemic’.

## OBSERVATION

Biofilms are key to the chronicity, recurrence, and recalcitrance-to-treatment of greater than 80% of bacterial infections owing, in part, to a protective underlying extracellular DNA (eDNA)-dependent matrix (1). Given the canonical and multifaceted ability of biofilms to resist eradication, novel approaches are urgently needed. After release from biofilm residence via various methodologies, bacteria exhibit a unique phenotype of greatly increased sensitivity to antibiotics (2–4). We reported this newly released (NRel) phenotype after disruption of diverse biofilms by a monoclonal antibody directed at protective domains of the ubiquitous bacterial DNABII proteins, IHF and HU (5), which maintain the structural integrity of the eDNA-dependent extracellular matrix. DNABII-directed monoclonal antibody-induced NRel of nontypeable *Haemophilus influenzae* (NTHI), methicillin-resistant *Staphylococcus aureus* (MRSA), non-tuberculous *Mycobacteria* (NTM), all seven ESKAPEE pathogens, *Burkholderia cenocepacia,* and *Streptococcus pneumoniae* are significantly more sensitive to antibiotics (6, 7). In studies of NTHI-induced otitis media in chinchillas, *Aggregatibacter actinomycetemcomitans*-induced peri-implantitis in rats, and *Pseudomonas aeruginosa-*induced murine lung infection (8–10), *in situ* biofilms were similarly rapidly eradicated with concomitant disease resolution without use of antibiotics, which led us to wonder whether NRel were also more vulnerable to killing by innate immune effectors.

Thereby, we investigated the relative sensitivity of anti-DNABII-induced NTHI NRel to killing by human PMNs and reported significantly augmented killing linked to their diminished ability to mediate oxidative stress (11). Here, we assayed killing of three clinical isolates of both MRSA and NTHI as model human respiratory tract pathogens (12, 13) by antimicrobial peptides (AMPs) given their essential role in first-line defense against infection (14, 15). AMPs chosen were human beta defensins 1 and 3 and the cathelicidin LL-37 as they are commonly expressed within the respiratory tract or by PMNs.

**Fig 1:**
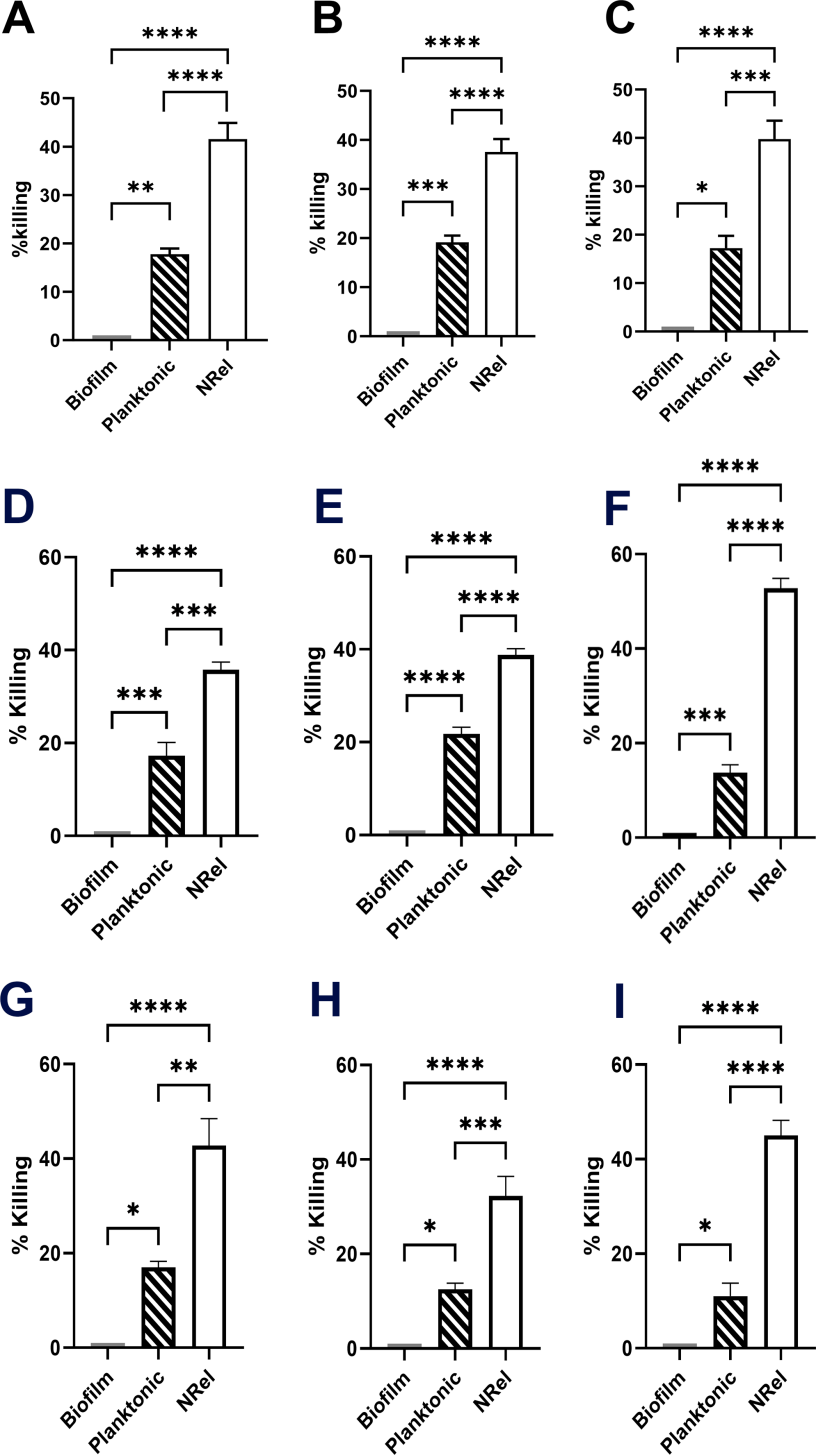
Relative killing of MRSA subpopulations by hBD-1, hBD-3, or LL-37. MRSA that was either biofilm-resident; grown planktonically to mid-log phase, or newly released from biofilm residence (NRel) by incubation with MsTipMab for 2 h, then incubated for 1.5 h with either (A, D, and G) 10 µg hBD-1; (B, E, and H) 5 µg hBD-3, or (C, F, and I) 0.3 µg LL-37 per mL of DIS. Relative killing of MRSA clinical isolates recovered from a person with cystic fibrosis (A, B, and C), from a bone abscess (SAS130, panels D, E, and F) and an invasive blood isolate (SAS135, panels G, H, andI). AMPs were used at physiologically relevant concentrations (16–18) and at a concentration pre-determined to restrict killing of planktonically grown MRSA to ≤20% to allow detection of any enhanced ability to kill NRel. Note significantly increased ability of all three tested AMPs to kill anti-DNABII MRSA NRel (*P* ≤ 0.0001).

**Fig 2:**
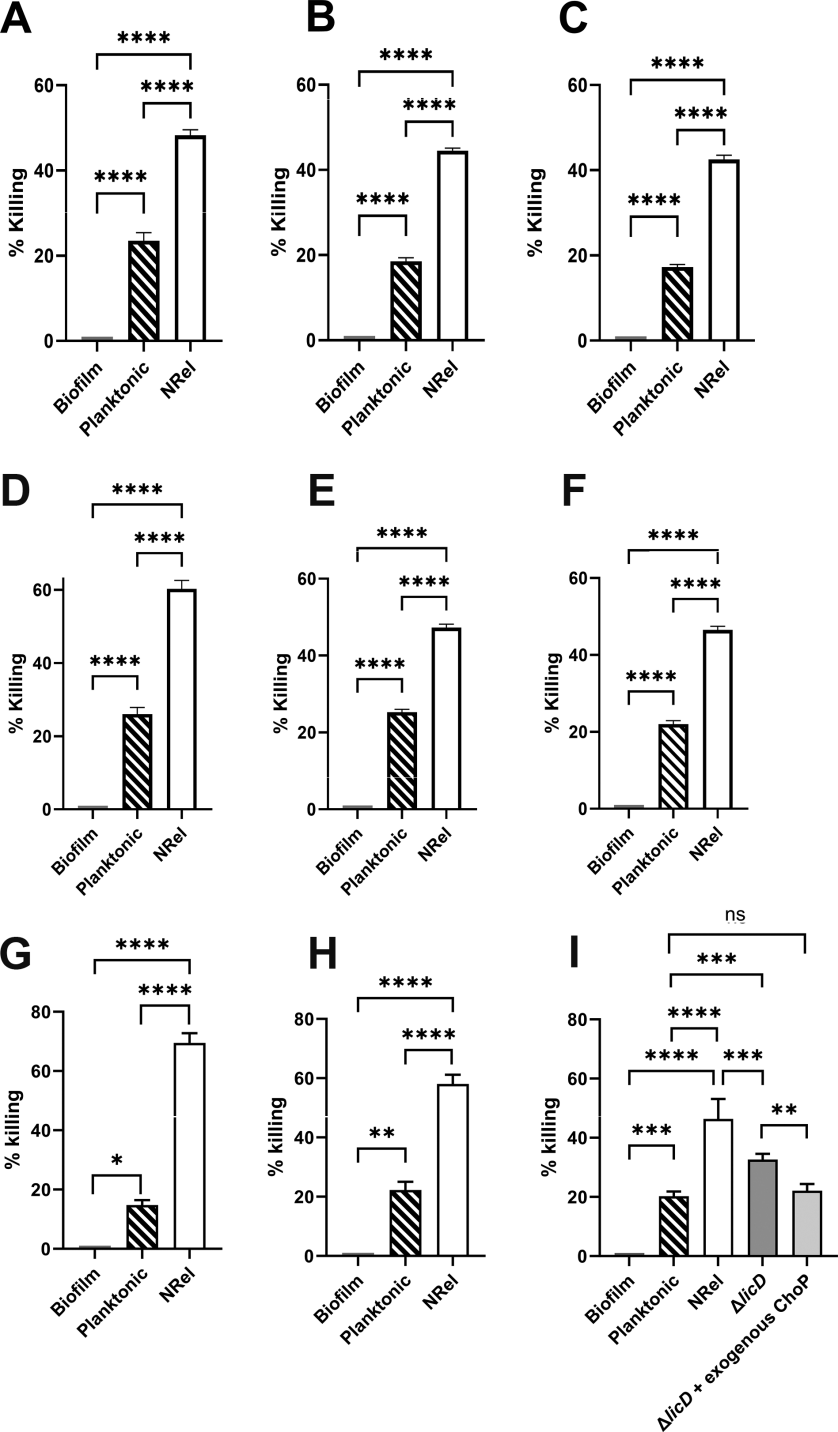
Relative killing of NTHI isolates by hBD-1, hBD-3, or LL-37. AMPs were used at physiologically relevant concentrations (16–18) and at a concentration pre-determined to restrict killing of planktonically grown NTHI ≤20% to allow detection of any enhanced ability to kill NRel. NTHI #1128 that was either biofilm-resident, incubated planktonically to mid-log phase or been newly released from biofilm residence (NRel) by incubation with MsTipMab were then incubated for 1 h with either (**A**) 10 µg hBD-1, (**B**) 5 µg hBD-3, or (**C**) 0.3 µg LL-37. The same three populations of NTHI #1728 were treated similarly with either (**D**) 10 µg hBD-1, (**E**) 5 µg hBD-3, or (**F**) 0.3 µg LL-37. Finally, we assessed the same three populations of NTHI #86-028NP following treatment with either (**G**) 10 µg hBD-1, (**H**) 5 µg hBD-3, or (**I**) 0.3 µg LL-37. Note significantly increased ability of all three tested AMPs to kill all three anti-DNABII NTHI NRel populations (*P* ≤ 0.0001). To begin to investigate the role of surface charge in sensitivity to killing by LL-37, we tested an isogenic ∆*licD* mutant of NTHI strain #86-028NP both with and without the addition of exogenous ChoP (**I**). Note significantly greater killing of the ∆*licD* mutant by LL-37 than the planktonically grown parental isolate (*P* ≤ 0.001), which was significantly reduced (*P* ≤ 0.005) and also restored back to that of the parental isolate by the addition of exogenous ChoP. Interestingly, anti-DNABII NTHI NRel were significantly more sensitive to LL-37 killing than the ∆*licD* mutant (*P* ≤ 0.0005). Collectively, these data suggested a role of outer membrane charge, as well as likely other factors, in the ability of LL-37 to kill anti-DNABII NTHI NRel.

Relative killing of biofilm-resident, mid-log planktonic or NRel of both MRSA and NTHI revealed the expected resistance of biofilm-resident MRSA to AMP killing; however, anti-DNABII MRSA NRel from all three clinical isolates were significantly more sensitive to killing by hBD-1, hBD-3, and LL-37 than even when planktonically grown (Fig. 1A through I) (*P* ≤ 0.0005). Similarly, biofilm-resident NTHI resisted AMP killing; however, killing of anti-DNABII NTHI NRel was significantly greater than that of either the biofilm-resident or planktonic subpopulations (Fig. 2A through I) (*P* ≤ 0.0001) for all three clinical NTHI isolates.

Cationic AMPs preferentially target the negatively charged bacterial cell surface (19, 20). As such, NTHI decorates its outer membrane with phosphorylcholine (ChoP; product of *lic*D expression) to reduce the net negative charge (21, 22). Earlier, we reported a 5.7-fold decreased LicD abundance in anti-DNABII NTHI NRel versus planktonically grown (5). To assess whether relative surface charge contributed to the observed LL-37-mediated killing of NTHI, we tested its ∆*licD* mutant, which was significantly greater than that of the planktonically grown parent (*P* ≤ 0.001) and mitigated by addition of 5 µg exogenous ChoP (Fig. 2I). Interestingly, however, killing of NRel was greater than that of *∆licD* (*P* ≤ 0.0001), which suggested additional mediator(s) of vulnerability and likely their reported increased membrane permeability (11).

Biofilms contribute significantly to the recalcitrance of the majority of chronic and recurrent bacterial diseases to treatment, necessitating urgent development of novel strategies. Clinical use of AMPs has been considered (23); however, there are challenges to this approach (24). As such, we have been exploring the potential clinical advantage of leveraging the anti-DNABII-mediated NRel phenotype for improved medical management of recalcitrant biofilm infections. The release of bacteria from the biofilm into the NRel state does not kill the bacteria, as we have shown previously by viability counts (see Fig. 3 in Goodman et al. [25] and Fig. S5 in Brockson et al. [26]). However, these newly released bacteria have a highly susceptible phenotype. Whereas the NRel phenotype is indeed transient (2–5), this phenotypic vulnerability nonetheless likely offers a unique many hours long window for clinical intervention. Delivery of humanized DNABII-directed monoclonal, currently in clinical evaluation (NCT05629741 and NCT06159725), to a patient is hoped to release pathogenic biofilm bacteria into the highly vulnerable NRel state for rapid clearance by host innate immune effectors, or if needed, traditional antibiotics that are ineffective against biofilms but effectively kill NRel. Collectively, these data add to others that strongly suggest that the NRel phenotype can be leveraged clinically to both mediate rapid disease resolution while simultaneously limiting further contribution to the global AMR crisis (27).

Nontypeable *Haemophilus influenzae* (NTHI) strain #86-028NP (28) from the nasopharynx of a child with chronic otitis media or its ChoP-deficient mutant Δ*licD* along with clinical NTHI strains 1128 and 1728 recovered from the middle ears of children with chronic otitis media was grown in Brain Heart Infusion broth supplemented (sBHI) with hemin (2 µg/mL) (Sigma-Aldrich, Cat no. H9039) and β-NAD (2 µg/mL) (Sigma-Aldrich, Cat no. N1511), whereas a clinical isolate of methicillin-resistant *Staphylococcus aureus* (MRSA) (recovered from a child with cystic fibrosis), an invasive MRSA isolate (SAS130) recovered from a child with a bone abscess, and MRSA isolate SAS135, an invasive and persistent isolate recovered from the blood, were grown on Tryptic Soy agar (TSA) or broth (TSB), all at 37°C with 5% CO_2_ in a humidified atmosphere.

Lyophilized LL-37 (Rockland Immunochemicals, Inc., Pottstown, PA) or human β-defensin 1 (hBD-1) (GenScript Biotech, Piscataway, NJ) was reconstituted in sterile deionized water and stored at −20°C. Carrier-free recombinant human β-defensin 3 (hBD-3) (R&D Systems, Minneapolis, MN) was reconstituted in sterile 4 mM HCl. Phosphocholine chloride sodium salt hydrate (98.0+% [phosphorylcholine, ChoP], TCI America) was dissolved immediately prior to use in 10 mM sodium phosphate buffer (pH 7.2).

One colony of NTHI or MRSA was inoculated into equilibrated chemically defined RPMI-based medium (DIS) (supplemented with 2 µg hemin/mL for NTHI only, 0.07 mM CaCl_2_, and 0.7 mM MgSO_4_) (29, 30) and grown to mid-log phase by static incubation for 2.5 or 3 h for MRSA or NTHI, respectively. DIS sustains bacterial viability without inhibiting the action of AMPs (30).

NTHI or MRSA (2.5 mL at 2  ×  10^5^ CFU/mL) was seeded into 10 cm^2^ flat tissue culture tubes (TPP, Trasadingen, Switzerland, Cat no. 91243) and allowed to form biofilms for 16 h, after which tubes were carefully inverted and medium that contained non-adherent bacteria was poured off. While inverted, 2.5 mL equilibrated (37°C, 5% CO_2_) Dulbecco’s phosphate buffered saline without calcium or magnesium (DPBS) was added. Tubes were rotated 360° to gently remove additional non-adherent bacteria, and DPBS was poured off. To generate α-DNABII NRel, washed biofilms were incubated for 2 h with either DIS medium alone or with DIS that contained 5 µg/0.8 cm^2^ murine monoclonal directed against the protective tip domains of a DNABII protein (called MsTipMab) (10). NRel were quantified by serial dilution of the fluids recovered from above the biofilm after 2 h.

Mid-log phase planktonic or NRel of MRSA or NTHI were collected, subjected to gentle water bath sonication to disrupt aggregates (11), then diluted to 1 × 10^8^ CFU/mL DIS. Diluted cultures were sonicated with 10 µL each added into wells of a sterile Sigmacote-siliconized polystyrene 96-well plate. Ninety microliters of AMP dissolved in 10 mM sodium phosphate buffer or buffer alone (growth control) was added to the wells. AMPs were diluted to a concentration pre-determined to limit planktonic killing to 10%–20% to facilitate detection of any enhanced NRel killing. Assay plate was incubated for 1 or 1.5 h for NTHI and MRSA, respectively. For killing of biofilm-resident bacteria, 16 h biofilms were incubated with AMPs or buffer alone prior to washing, then biofilms recovered as previously reported (6). Percent killing was determined as [(CFU/mL growth control – CFU/mL bacteria mixed with AMP)/CFU/mL growth control] × 100% ± SD. Assays were repeated three times on separate days.

For studies of the role of ChoP in relative sensitivity to killing by LL-37, buffer alone or ChoP in 10 µL of 10 mM sodium phosphate buffer was added to each well of a sterile siliconized 96-well plate, followed by 10 µL of mid-log phase planktonic NTHI #86-028NP or *ΔlicD*, then 80 µl of LL-37 or buffer alone; relative killing determined as above.

## Data Availability

All relevant data are contained within the manuscript and its supporting information files.
